# A machine learning and nomogram-based study: effect of applying biologically formulated platelet-rich plasma (PRP) on the degree of pain relief after rotator cuff repair and prediction modeling, integrating biomedicine and artificial intelligence

**DOI:** 10.3389/fmed.2025.1647551

**Published:** 2025-10-08

**Authors:** Jianguo Zhang, Jian Gao, Haoyu Feng, Wei Liu

**Affiliations:** Shanxi Bethune Hospital, Shanxi Academy of Medical Sciences, Third Hospital of Shanxi Medical University, Tongji Shanxi Hospital, Taiyuan, China

**Keywords:** machine learning, nomogram, platelet-rich plasma (PRP), rotator cuff injuries, pain relief

## Abstract

**Introduction:**

Rotator cuff repair, a common orthopedic surgery, often leads to considerable postoperative pain that delays functional recovery. Platelet-rich plasma (PRP) has been increasingly used as a biologically active autologous therapy to promote tendon healing and reduce inflammation, but its analgesic effects remain inconsistent across individuals. Conventional linear models may fail to account for complex patient-specific interactions such as age, body mass index (BMI), and preexisting inflammatory status.

**Methods:**

We developed a machine learning–based prediction model combined with a nomogram to assess the analgesic efficacy of PRP following rotator cuff repair. Clinical and demographic variables were incorporated to capture nonlinear relationships influencing pain reduction.

**Results:**

The machine learning framework demonstrated improved predictive accuracy compared with traditional models. The nomogram provided an interpretable and clinically applicable visualization of individualized pain-relief trajectories.

**Discussion:**

This study highlights the potential of integrating machine learning and nomogram approaches to enhance personalized prediction of PRP analgesic response. Such individualized forecasting tools may support tailored postoperative management strategies and optimize rehabilitation outcomes.

## Introduction

Rotator cuff tear is a common cause of shoulder dysfunction and chronic pain, particularly among middle-aged and older adults (1–4). Arthroscopic rotator cuff repair has become the standard treatment for full-thickness and symptomatic partial-thickness tears, aiming to restore shoulder function, relieve pain, and improve quality of life (5–8). Despite advances in surgical techniques and rehabilitation protocols, a significant proportion of patients continue to experience moderate to severe postoperative pain (9, 10), which can delay functional recovery, impair rehabilitation adherence, could might have a role in the development of chronic pain disorders. This emphasizes how urgently customized, effective postoperative pain control techniques are needed.

In recent years, platelet-rich plasma (PRP) has emerged as a promising biological adjunct in musculoskeletal surgery (11, 12). PRP is an autologous blood-derived product with a high concentration of platelets and a milieu of bioactive factors, including transforming growth factor-β (TGF-β), platelet-derived growth factor (PDGF), and vascular endothelial growth factor (VEGF) (13–17), which are involved in tissue regeneration, inflammation modulation, and analgesic responses. When applied intraoperatively or postoperatively, PRP has been shown to enhance tendon-to-bone healing and potentially reduce inflammation-related pain (18, 19). Because of these biological characteristics, PRP is a desirable option for enhancing results after rotator cuff surgery.

Clinical data on PRP’s effectiveness in reducing pain, however, is still conflicting. PRP has been shown in certain randomized controlled trials and meta-analyses to significantly reduce postoperative pain and speed up functional recovery, but other studies have found no discernible difference when compared to conventional care or a placebo (20–22). These contradictory results might be the result of variations in PRP preparation procedures, surgical methods, and application timing, as well as underlying patient heterogeneity. More significantly, the therapeutic value of PRP in individualized treatment planning is limited because it is rarely discussed in the literature today on which patients are most likely to benefit from it.

Even after controlling for relevant confounders, our initial multivariate regression analysis indicates that PRP administration is substantially related with higher postoperative pain alleviation. Furthermore, we found that a number of other factors, including age, smoking status, comorbidities, body mass index (BMI), baseline visual analog scale (VAS) scores, and the severity of rotator cuff damage, also have independent impacts on pain outcomes. Higher BMI patients, for instance, frequently have chronic inflammation and changed pain sensitivity, which may affect how they perceive pain in the first place as well as how they react to PRP therapy. The aforementioned results underscore the necessity of multivariate risk stratification instruments that surpass single-variable analysis.

Complex, non-linear interactions between several factors are difficult for traditional statistical models, like logistic regression, to capture. However, by automatically identifying latent patterns, non-linear correlations, and variable interactions that may not be immediately obvious with traditional methods, machine learning (ML) algorithms provide better performance in high-dimensional data settings.

While logistic regression is a classical supervised machine learning algorithm, it assumes linear relationships between predictors and the log-odds of the outcome, which may limit its ability to model complex non-linear interactions or higher-order variable dependencies without explicit feature engineering. In contrast, non-parametric machine learning methods such as random forests and gradient boosting can automatically detect latent patterns and non-linear interactions within high-dimensional data, often yielding improved predictive performance in complex clinical settings.

Additionally, combining ML-derived models with nomograms—graphical depictions of predictive models that calculate the likelihood of a clinical event—offers a potent way to generate personalized, easily comprehensible risk evaluations for physicians.

In this study, we used multivariate logistic regression—a widely used supervised machine learning algorithm—as the basis for nomogram construction due to its interpretability and clinical familiarity. We then compared its predictive performance with other advanced machine learning models to explore potential non-linear interactions that might not be captured by parametric approaches.

In order to provide individualized estimates of postoperative pain relief, we therefore set out to assess the effect of PRP application on postoperative pain outcomes after arthroscopic rotator cuff repair using real-world clinical data and create a machine learning-based nomogram prediction model that integrates PRP application with important clinical and demographic variables. By doing this, we want to close the gap between clinical application and biological justification by providing a decision-support tool to help surgeons create specialized perioperative pain management strategies and to direct patient selection for PRP therapy.

By combining clinical epidemiology, biological information, and artificial intelligence techniques, this multidisciplinary study advances the area of orthopedic precision pain management. The proposed model may ultimately enhance clinical decision-making, optimize resource allocation, and improve patient outcomes by identifying those who are most likely to benefit from PRP-based interventions.

## Materials and methods

In the PRP group, autologous PRP was prepared intraoperatively using a standard two-step centrifugation protocol. Whole blood (approximately 20 mL) was collected from each patient into citrate-containing tubes. The first centrifugation (soft spin) was conducted at 1,500 rpm for 10 min to separate plasma from red blood cells, followed by a second centrifugation (hard spin) at 3,500 rpm for 10 min to concentrate the platelets. The resulting PRP had a 4–5-fold increase in platelet concentration compared to baseline whole blood levels.

A total volume of 4–5 mL of PRP was injected intraoperatively at the tendon-to-bone interface around the repaired rotator cuff footprint under direct arthroscopic visualization, immediately following suture anchor placement and prior to wound closure. No additional PRP was administered postoperatively.

platelet-rich plasma was prepared using a standardized two-step centrifugation method: 1500 rpm for 10 min, followed by 3500 rpm for 10 min. The final PRP product was applied intraoperatively at the tendon–bone interface after rotator cuff repair.

Inclusion criteria were: age ≥18 years, complete baseline and follow-up data, and no prior shoulder surgery or systemic inflammatory disease. At 3 months after surgery, the main result was pain alleviation, which was defined as a decrease of at least 50% in the VAS score from the baseline.

A total of 240 patients (31 in the control group and 209 in the PRP group) were included in the analysis. Patients with incomplete baseline or follow-up data were excluded prior to analysis. No patients were lost to follow-up at the 3 months assessment, and there were no missing values for the included variables. Table 1 summarizes the baseline demographic and clinical characteristics of the control group and PRP group.

**TABLE 1:** Comparison of baseline demographic, clinical, and surgical characteristics between the control and platelet-rich plasma (PRP) groups.

Variable	Category	Control (*n*, %)	PRP (*n*, %)
Age	40–50	13 (41.9%)	72 (34.4%)
	<40	10 (32.3%)	63 (30.1%)
	>50	8 (25.8%)	74 (35.4%)
Sex	Female	16 (51.6%)	116 (55.5%)
	Male	15 (48.4%)	93 (44.5%)
Steatosis	No	18 (58.1%)	178 (85.2%)
	Yes	31 (41.9%)	31 (14.8%)
Smoke	No	22 (71.0%)	158 (75.6%)
	Yes	9 (29.0%)	51 (24.4%)
Diabetes	No	14 (45.2%)	104 (49.8%)
	Yes	17 (54.8%)	105 (50.2%)
Surgical type	Type 1	9 (29.0%)	72 (34.4%)
	Type 2	12 (38.7%)	64 (30.6%)
	Type 3	10 (32.3%)	73 (34.9%)
Rotator cuff	Medium	23 (74.2%)	149 (71.3%)
	Small	8 (25.8%)	60 (28.7%)
BMI	24–28	14 (45.2%)	76 (36.4%)
	<24	13 (41.9%)	92 (44.0%)
	≥28	4 (12.9%)	41 (19.6%)
VAS	0–3	8 (25.8%)	73 (34.9%)
	4–6	13 (41.9%)	69 (33.0%)
	7–10	10 (32.3%)	67 (32.1%)
ESR	High↑	9 (29.0%)	77 (36.8%)
	Mild↑	12 (38.7%)	67 (32.1%)
	Normal	10 (32.3%)	65 (31.1%)
CRP	High↑	13 (41.9%)	67 (32.1%)
	Mild↑	9 (29.0%)	70 (33.5%)
	Normal	9 (29.0%)	72 (34.4%)
ASES	High	13 (41.9%)	71 (34.0%)
	Low	7 (22.6%)	71 (34.0%)
	Medium	11 (35.5%)	67 (32.1%)

The VAS is a standard 0–10 scale used to evaluate pain intensity, where 0 represents “no pain” and 10 indicates “worst imaginable pain.” Patients completed VAS assessments preoperatively (within 1 week before surgery) and at the 3 months postoperative follow-up. Pain relief was defined as a ≥50% reduction in VAS scores compared to baseline.

Sex, age, BMI, smoking status, diabetes, baseline pain ratings, inflammatory markers (ESR, CRP), ASES scores, rotator cuff tear grade, surgical method, and PRP application status were among the clinical and demographic information gathered. Smoking history (“smoke”) was defined as having a history of tobacco use and continuing to smoke during the perioperative period, coded as 0 = no and 1 = yes.

These variables were extracted from electronic medical records. The ASES (American Shoulder and Elbow Surgeons) score ranges from 0 to 100, with higher scores reflecting better shoulder function. Inflammatory markers such as ESR and CRP were measured using standard laboratory procedures during preoperative clinical assessments.

For modeling purposes, sex was coded as a binary variable (0 = female, 1 = male). Smoking status and diabetes were also treated as binary variables (0 = no, 1 = yes).

Age and BMI were categorized based on clinically relevant thresholds. Age was divided into three groups: <40 years (coded as 1), 40–50 years (coded as 2), and >50 years (coded as 3). BMI was categorized according to standard classification: <24 kg/m^2^ (normal, coded as 1), 24–28 kg/m^2^ (overweight, coded as 2), and ≥28 kg/m^2^ (obese, coded as 3), following Chinese health guidelines.

Variable selection was first carried out using the least absolute shrinkage and selection operator (LASSO) regression with 10-fold cross-validation to find the best predictors in order to prevent overfitting and deal with multicollinearity. All variables were then subjected to univariate logistic regression, and those with *p* < 0.10 were then included in a multivariate logistic model in order to identify independent predictors. Statistically significant factors were then incorporated into a nomogram to visualize individual risk using the “rms” package in R. The performance of the nomogram was evaluated by the area under the receiver operating characteristic curve (AUC), concordance index (C-index), and calibration plots. Internal validation was performed via 1,000 bootstrap resamples.

The same dataset was used to train a number of machine learning models, including logistic regression, random forest (RF), support vector machine (SVM), extreme gradient boosting (XGBoost), and multilayer perceptron (MLP), in order to further evaluate the prediction’s resilience and find non-linear connections. Model performance was compared using AUC, sensitivity, specificity, and total accuracy using 5-fold cross-validation.

Other continuous variables, including baseline VAS score, ESR, CRP, and ASES score, were used in their original scale without transformation or grouping.

**FIGURE 1:**
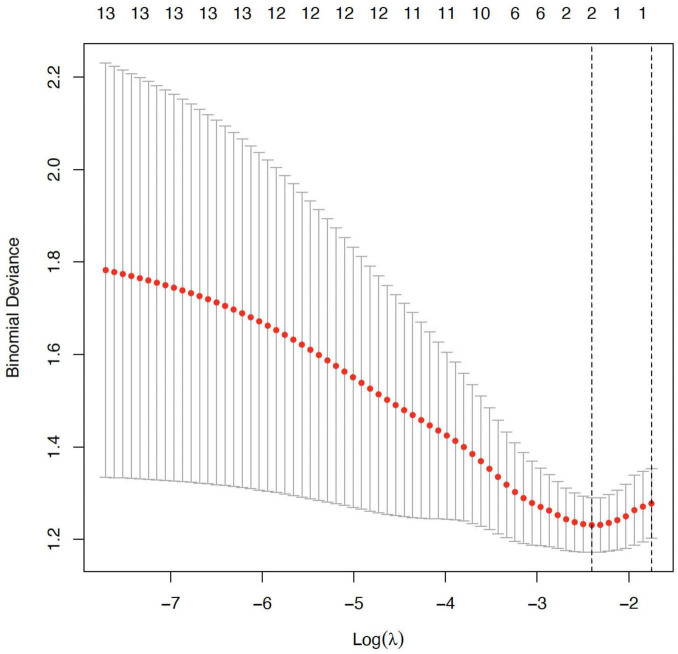
Least absolute shrinkage and selection operator (LASSO) coefficient profile plot.

This Figure 1 shows the LASSO coefficient profiles of all candidate predictors as a function of the logarithmic value of the regularization parameter λ. As λ increases (moving left to right), more coefficients shrink toward zero, indicating regularization strength. When log(λ) is sufficiently high, only a few variables remain with non-zero coefficients. This penalization process helps eliminate redundant or weakly associated variables. In our analysis, 13 variables initially entered the model, but as λ increased, only six variables retained non-zero coefficients, suggesting their stronger association with the outcome of postoperative pain relief.

**FIGURE 2:**
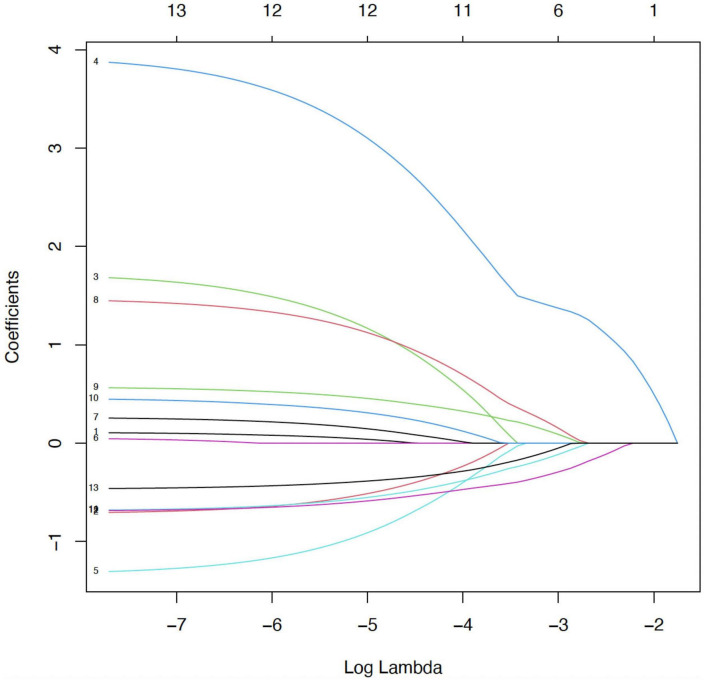
Selection of optimal λ via cross-validation.

The 10-fold cross-validation error (binomial deviation) curve for various λ values is shown in this graphic. The ideal balance between model complexity and prediction error is indicated by the vertical dotted line on the left, which shows the value of λ that minimizes the cross-validated error (λ_min). Six variables in all were chosen for additional modeling at this ideal λ. We chose λ_min to optimize predictive information, even if the right vertical line (optional λ_1se) is more conservative and contains fewer variables. The optimal value of λ was determined through 10-fold cross-validation, as illustrated in Figure 2, which shows the binomial deviation curve and the corresponding λ values.

**FIGURE 3:**
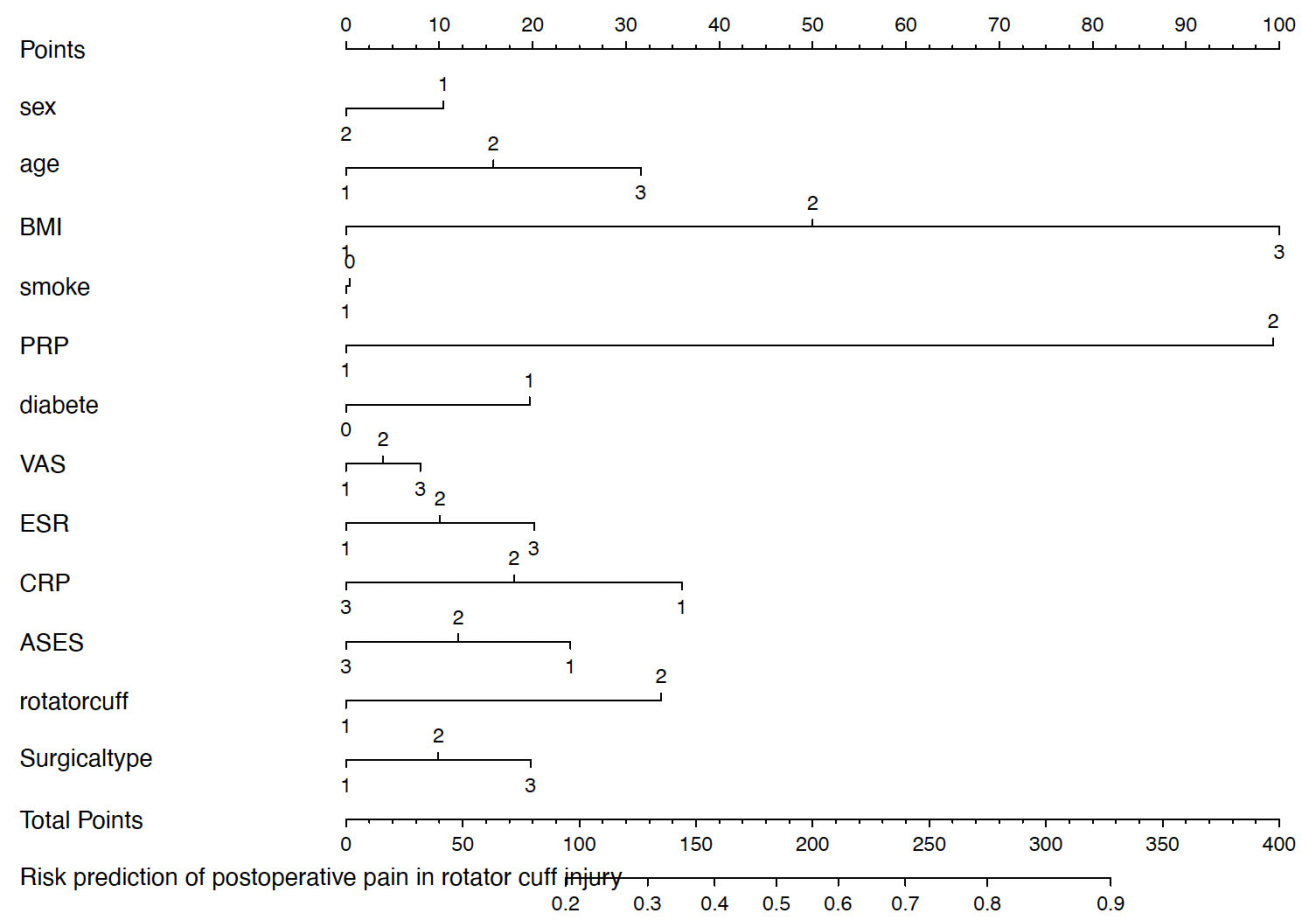
Nomogram for predicting the probability of postoperative pain relief in rotator cuff injury patients. Predictors include sex (0 = female, 1 = male), age group (<40, 40–50, >50 years), body mass index (BMI: <24, 24–28, ≥28 kg/m^2^), smoking history (0 = no, 1 = yes), diabetes (0 = no, 1 = yes), baseline VAS (Visual Analog Scale, 0–10), inflammatory markers [ESR, C-reactive protein (CRP)], functional status [acromioclavicular function score (ASES) score, 0–100], rotator cuff tear classification, surgical type, and PRP application (0 = no, 1 = yes). The total score corresponds to the estimated probability of achieving ≥50% reduction in visual analog scale (VAS) score at 3 months postoperatively.

This nomogram Figure 3 was created using multivariate logistic regression to calculate each person’s unique likelihood of experiencing considerable postoperative pain alleviation 3 months after arthroscopic rotator cuff surgery, which is defined as a reduction of at least 50% in VAS score. Sex, age, body mass index (BMI), smoking status, PRP application, diabetes, baseline pain (VAS), inflammatory markers (ESR and CRP), functional status (ASES score), rotator cuff tear classification, and surgery type are among the many clinically significant indicators that are integrated.

A point scale representing each variable’s relative contribution to the final forecast is aligned with it at the top. At the bottom of the nomogram, the total score represents the anticipated likelihood of postoperative pain alleviation. Clinicians can add up the points allotted to each predictor based on the patient’s values.

The use of platelet-rich plasma, or PRP, had the most impact on postoperative results out of all the predictors. PRP recipients were given noticeably higher point values than non-receivers, suggesting a robust positive correlation with pain alleviation. This corroborates the findings of our multivariate analysis, which showed that PRP was an independent protective factor for postoperative pain, emphasizing its analgesic effectiveness in relation to inflammatory management and tendon repair.

On the other hand, in line with established biological and metabolic obstacles to healing, a greater body mass index and higher preoperative pain or inflammatory scores (such as ESR or CRP) tended to lower the likelihood of postoperative pain reduction.

In order to facilitate individualized decision-making concerning pain treatment techniques and the choice of PRP candidates for rotator cuff surgery, this nomogram offers a simple, interpretable clinical tool.

Feature selection was initially performed using LASSO regression to reduce dimensionality and identify variables with the strongest association with postoperative pain relief. Six predictors with non-zero coefficients at the optimal λ were selected.

However, to enhance the clinical interpretability and retain variables with established relevance in the literature and practice (e.g., sex, smoking status, surgical method), we incorporated additional clinically meaningful variables into the multivariate logistic regression model used for nomogram construction.

This hybrid approach aimed to balance statistical parsimony with clinical utility. To mitigate potential overfitting, we performed internal validation using 1,000 bootstrap resamples and evaluated model calibration and discrimination in both training and validation cohorts.

**FIGURE 4:**
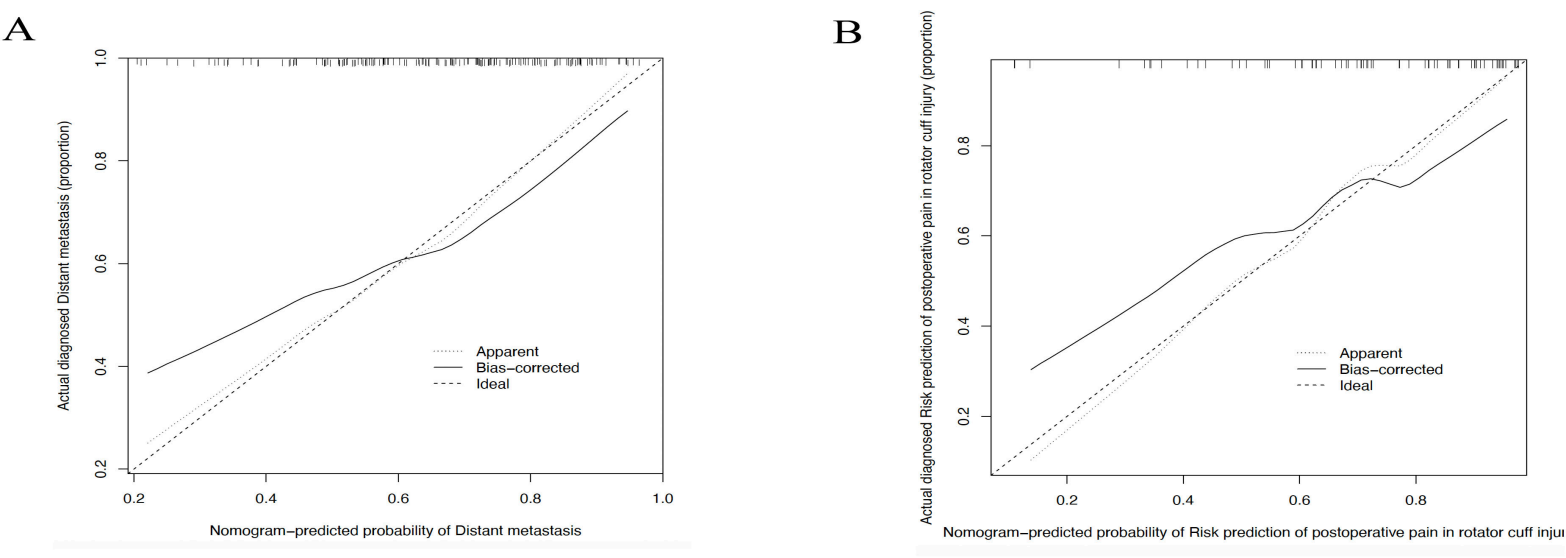
Calibration curves of the nomogram for predicting postoperative pain relief after rotator cuff injury. **(A)** Calibration curve in the modeling cohort; **(B)** Calibration curve in the validation cohort.

The calibration curve for the modeling population, which is utilized to assess the nomogram’s predictive ability in predicting postoperative pain alleviation following rotator cuff injury, is shown in Figure 4A. Three curves are shown on the chart: the bias-corrected curve (solid), the apparent curve (dotted), and the ideal line (dashed). In the 0.2–0.5 range, where the observed incidence of pain alleviation grows steadily with the projected values, the bias-corrected curve closely resembles the ideal line over the whole range of predicted probability (0.2–1.0). Even though there are slight variations in the 0.5–1.0 range, they stay within reasonable bounds, suggesting that the model exhibits decent calibration and prediction accuracy in the training population. This suggests that the nomogram can reliably estimate the probability of postoperative pain relief and has potential for clinical application in guiding prognosis and treatment planning.

The calibration curve for the validation population is displayed in Figure 4B. The bias-corrected curve shows overall agreement with the ideal line over the anticipated probability range (0.2–1.0), much as the modeling cohort. The curve closely resembles the ideal trend in the low to mid-range (0.2–0.6), however, there are minor variations that are still within a suitable range in the mid-to-high range (0.6–0.8). The curve progressively moves back toward the ideal line as the anticipated probability rises over 0.8. These results show that the model retains satisfactory calibration performance in the external validation cohort following adjustment. The nomogram’s generalizability and robustness are supported by the overall predictive agreement, notwithstanding the possibility of occasional overestimation or underestimating in higher probability ranges. Further refinement of the model, such as incorporating additional clinical predictors, may enhance its accuracy in complex scenarios.

Both the training and validation groups underwent receiver operating characteristic (ROC) curve analysis to assess the nomogram’s discriminatory capacity in forecasting postoperative pain reduction following rotator cuff surgery.

**FIGURE 5:**
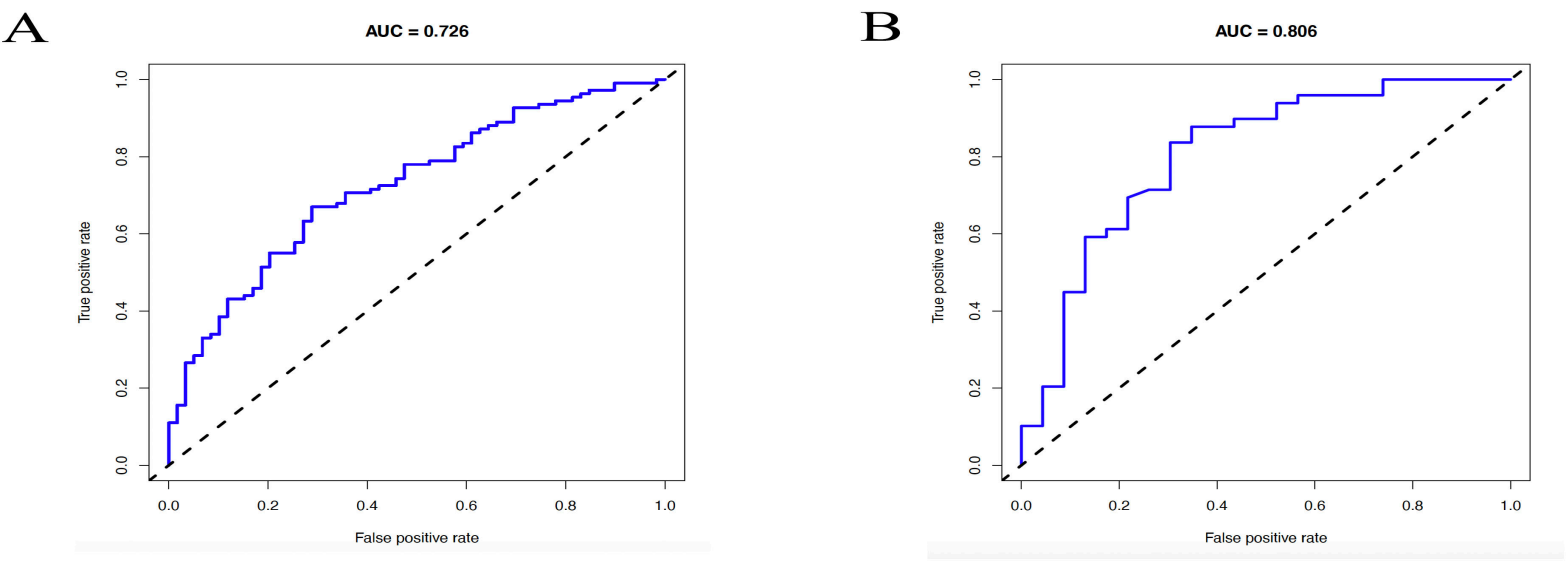
Receiver operating characteristic (ROC) curves of the nomogram for predicting postoperative pain relief following rotator cuff repair. **(A)** ROC curve in the training cohort, with an s area under the curve (AUC) of 0.726. **(B)** ROC curve in the validation cohort, with an AUC of 0.806.

A reasonable degree of discriminating was shown by the nomogram’s area under the ROC curve (AUC) of 0.726 in the training cohort, as seen in Figure 5A. The AUC rose to 0.806 in the validation cohort (Figure 5B), indicating that the nomogram performed satisfactorily when applied to a separate dataset and exhibiting strong prediction accuracy.

These results support the clinical utility of the model in stratifying patients based on the likelihood of achieving postoperative pain relief.

**FIGURE 6:**
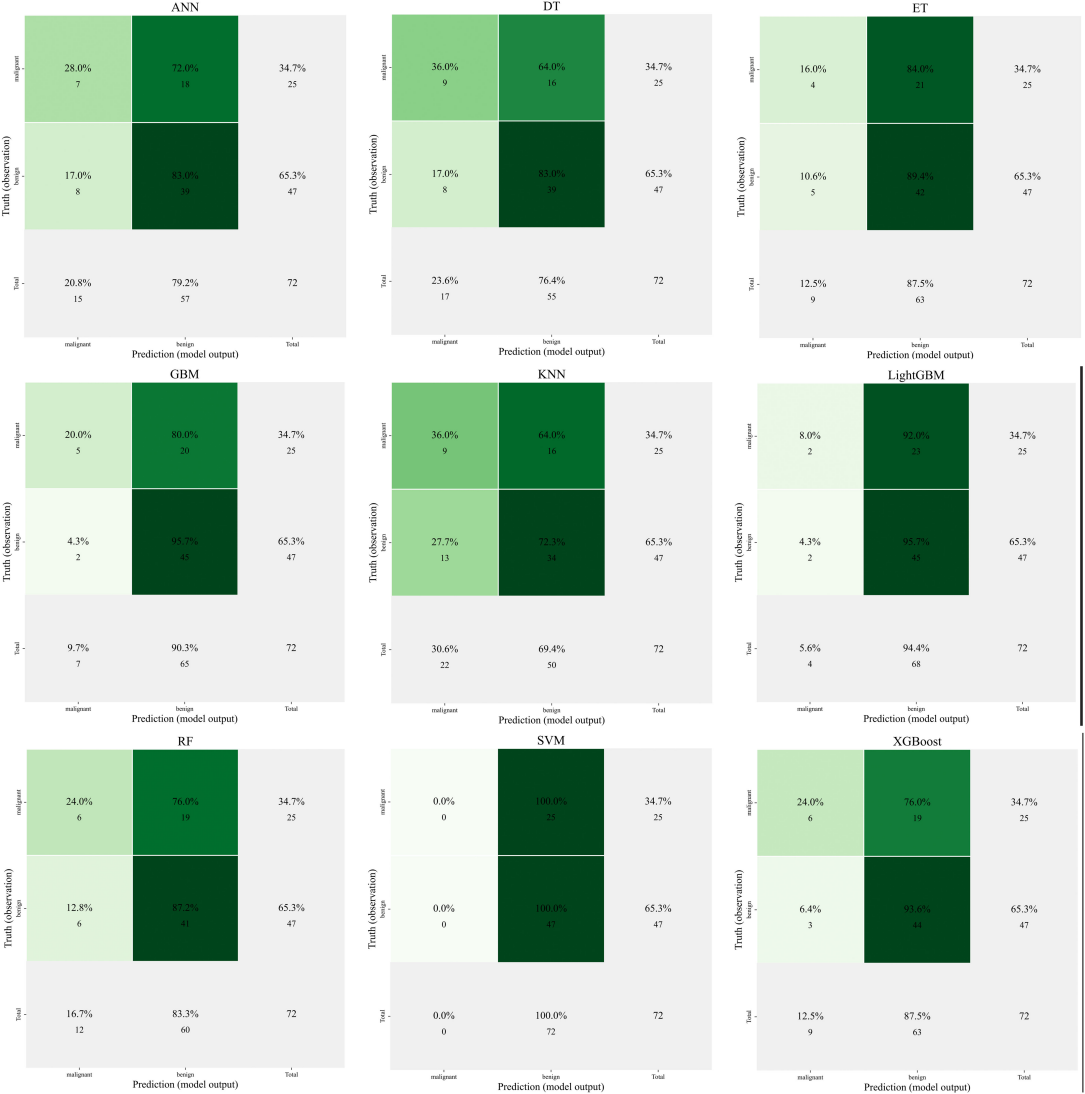
Comparison of nine machine learning models for predicting clinical outcomes.

Artificial neural networks (ANN), decision trees (DT), extra trees (ET), gradient boosting machines (GBM), K-nearest neighbors (KNN), LightGBM, random forests (RF), support vector machines (SVM), and XGBoost are among the nine popular machine learning techniques used in predictive modeling that are shown in Figure 6. These techniques are used for clinical feature-related classification, and model performance is assessed. The prediction accuracy, AUC value, and computed error of each model show how differently they perform.

The image illustrates how some models, including as XGBoost and Random Forest, perform better in terms of classification accuracy and AUC (area under the curve), highlighting their benefits when handling high-dimensional data and intricate non-linear interactions.

Unlike traditional nomograms (based on statistical regression analysis), which often assume linear correlations between variables, machine learning models may automatically capture complicated interaction effects in the data. Nomograms are easily interpreted, but machine learning techniques—particularly tree-based techniques like XGBoost and RF—offer more promise in terms of precision and applicability.

XGBoost has shown excellent performance in several experiments, but lacks the intuitive interpretability of nomogram. In contrast, SVMs and LightGBMs perform somewhat less well, but still provide valuable predictive results for specific tasks.

**FIGURE 7:**
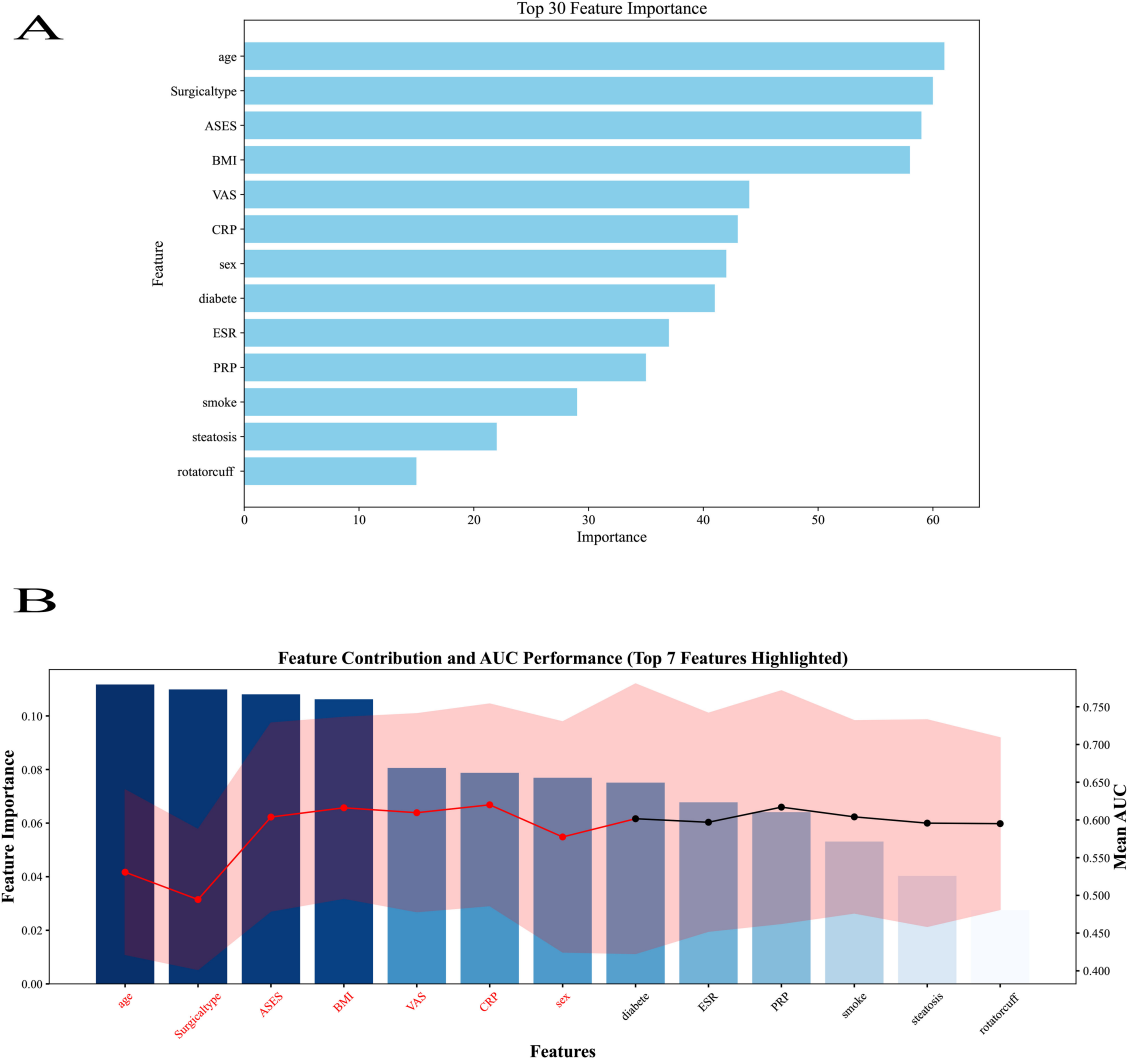
Feature importance ranking from the LightGBM model. **(A)** Top 13 predictors ranked by relative importance, with thicker bars indicating greater contribution to the model’s prediction. **(B)** Feature-wise classification performance showing mean AUC score. A total of 13 predictors were included: age group (<40, 40–50, >50 years), sex (0 = female, 1 = male), BMI group (<24, 24–28, ≥28 kg/m^2^), smoking history (0 = no, 1 = yes), diabetes (0 = no, 1 = yes), baseline visual analog scale (VAS) score (0–10), ESR, C-reactive protein (CRP), acromioclavicular function score (ASES) functional score (0–100), rotator cuff tear grade, surgical type, and, platelet-rich plasma (PRP) application (0 = no, 1 = yes). Bars represent the relative contribution of each variable to model classification performance.

The top 13 characteristics from a trained LightGBM model are displayed in Figure 7A in order of relevance; thicker bars denote a larger relative contribution to the model’s prediction. The feature-wise classification performance is shown in Figure 7B, where the mean AUC score and 95% CI for each feature were calculated by modeling it separately. Red highlights the top 13 traits with the greatest AUC values, indicating their potent discriminative power. When combined, the two panels offer contrasting viewpoints for finding reliable predictors in ensuing machine learning modeling: model-derived significance and standalone classification usefulness.

In the LightGBM model, all available predictors (*n* = 13) were ranked by relative importance, with BMI, diabetes, and baseline ASES score among the top-ranked variables. PRP application also appeared within the top tier of predictors, although it did not have the highest relative importance in this specific algorithm. It should be noted that feature importance rankings may vary between algorithms due to differences in calculation methods, and the LightGBM ranking reflects only one model’s perspective. Across multiple models, including logistic regression and SHAP analyses, PRP consistently emerged as a statistically significant and clinically relevant predictor of postoperative pain relief.

**FIGURE 8:**
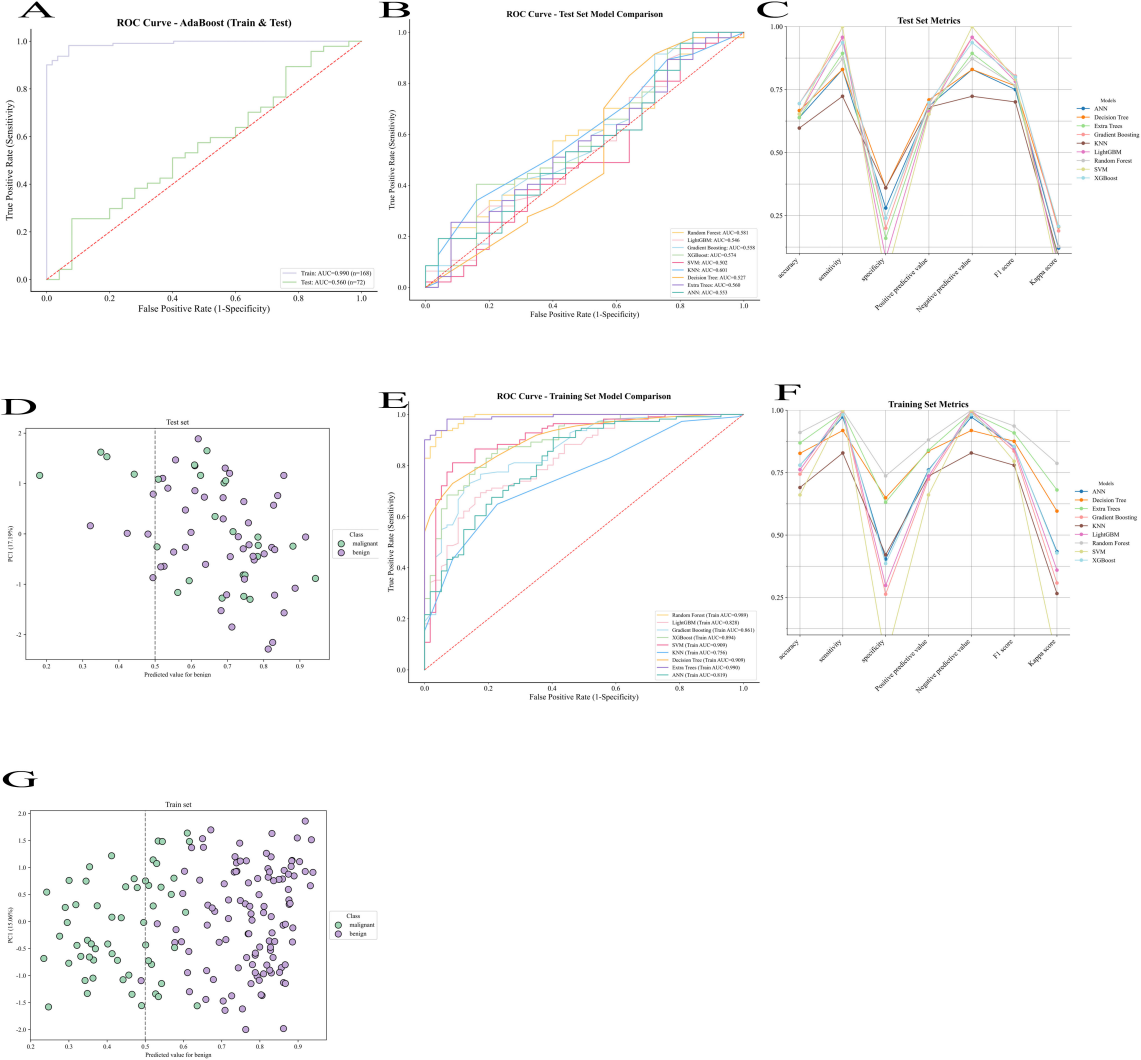
Comparison of machine learning models for predicting postoperative pain relief after rotator cuff repair. **(A)** Receiver operating characteristic (ROC) curve of AdaBoost model in the training and test sets. **(B)** ROC curves of multiple machine learning models in the test set. **(C)** Performance metrics (accuracy, sensitivity, specificity, etc.) of different models in the test set. **(D)** Principal component analysis (PCA) distribution of predicted values in the test set. **(E)** ROC curves of multiple machine learning models in the training set. **(F)** Performance metrics of different models in the training set. **(G)** PCA distribution of predicted values in the training set.

The AdaBoost model’s efficacy in predicting pain reduction following rotator cuff injury is illustrated by the ROC curves in Figure 8A. The X-axis shows the false positive rate (1-specificity), while the Y-axis shows the true positive rate (sensitivity). With an AUC of 0.990, the training set (purple curve) shows very high prediction accuracy, suggesting that the model fits the training data well; the test set (green curve) shows an AUC of 0.560, suggesting that the model generalizes poorly on the test data and that overfitting may be an issue. This discrepancy between the training and test set performance indicates potential overfitting of the AdaBoost model. The AdaBoost model showed a high training AUC (0.990) but a low test AUC (0.560), indicating potential overfitting. This is likely due to its sensitivity to noise and small sample sizes, suggesting that boosting may not be optimal for this clinical dataset. Future optimization might include parameter tuning, feature selection, or ensemble approaches to mitigate overfitting risks. The stochastic classifier’s performance is shown by the red dashed line. The figure shows that although the AdaBoost model performs well on the training set, it has limited generalization ability on new data, and further optimization of the model is needed to improve its prediction ability on unknown data.

The performance of many models on the test set is displayed in Figure 8B as various colored curves, each of which represents the model’s ROC curve and associated AUC values. Among the models are Artificial Neural Networks (ANN), Random Forest, LightGBM, Gradient Boost, XGBoost, Support Vector Machine (SVM), K Nearest Neighbors (KNN), Decision Trees, and Extra Trees. The rate of true positives (sensitivity) is shown on the y-axis, while the rate of false positives (1-specificity) is shown on the x-axis. The random classifier’s reference line is shown by the red dashed line. The difference in each model’s predictive capacity is shown by the AUC values, which range from 0.502 (SVM) to 0.601 (KNN), with SVM doing badly and KNN performing best on the test set.

The performance of the various models on the training set is displayed in Figure 8E as curves of various colors, each of which represents a model’s AUC value. The training set’s AUC values, which range from 0.756 (KNN) to 0.990 (Extra Trees), are likewise usually high, much like the test set model comparison graph. Strong fit is demonstrated by the Random Forest, XGBoost, and Extra Trees models, which perform better on the training set. The random classifier’s baseline is also shown by the red dashed line. Even if all models perform well on the training set, this figure demonstrates that a major problem with them is still their inability to generalize to the test set.

These ROC curve comparisons help us to evaluate the performance of different machine learning models in the prediction of pain relief after rotator cuff injury, reflecting the differences in the classification ability of each model and its performance on training and test data.

The accuracy, sensitivity, specificity, positive and negative predictive values, F1 score, and Kappa coefficient of the various machine learning models on the test set are displayed in Figure 8C. Different colored lines represent each model’s performance, with ANNs (Artificial Neural Networks) outperforming the others in a number of parameters, particularly sensitivity and positive predictive value, which are both comparatively high. In contrast, SVM and XGBoost exhibit subpar performance on these criteria. Overall, there was a significant range in the models’ performance metrics across the test set, suggesting that the models’ performance was inconsistent across many criteria.

The distribution of the test set samples’ projected values using Principal Component Analysis (PCA) is displayed in Figure 8D. The Y-axis shows the value of the first principal component (PC1), while the X-axis shows the expected values for benign (benign). Purple dots indicate benign samples, whereas green dots indicate malignant (malignant) samples. The categorization boundaries are shown by the dashed lines. There is a clear distinction between the anticipated values of benign and malignant samples, as seen by the distribution in the figure, which displays the model’s discriminating in predicting benign and malignant samples.

Figure 8F shows the performance of different machine learning models on the training set, with metrics such as accuracy, sensitivity, and specificity. Compared to the performance of the models on the test set, the models on the training set usually perform better, with generally higher AUC values. Extra Trees and ANN perform the best on the training set, while the other models such as Decision Trees and SVMs are relatively weaker. This figure reflects the adaptability of each model on the training set, but also suggests the need to focus on generalization ability to avoid overfitting.

Figure 8G this figure is similar to Figure 8F and shows the predictive value of the training set samples in relation to principal component analysis. The green dots indicate malignant samples and the purple dots indicate benign samples. x-axis indicates the predicted benign values and y-axis indicates the first principal component (PC1). This suggests that the model is better at differentiating between the training set’s categories.

In order to assist clinicians in forecasting postoperative pain relief based on patient characteristics and to provide decision support for individualized treatment and care, we developed a machine learning model in this study for predicting pain relief in patients following rotator cuff injuries.

We interpreted and examined the model’s prediction findings using SHAP values (Shapley Additive Explanations). A machine learning model may be interpreted using SHAP values, which provide us insight into how each feature contributes to the final prediction outcomes. This allows us to further improve the algorithm and clearly show which features are most crucial for predicting pain alleviation following rotator cuff injuries.

**FIGURE 9:**
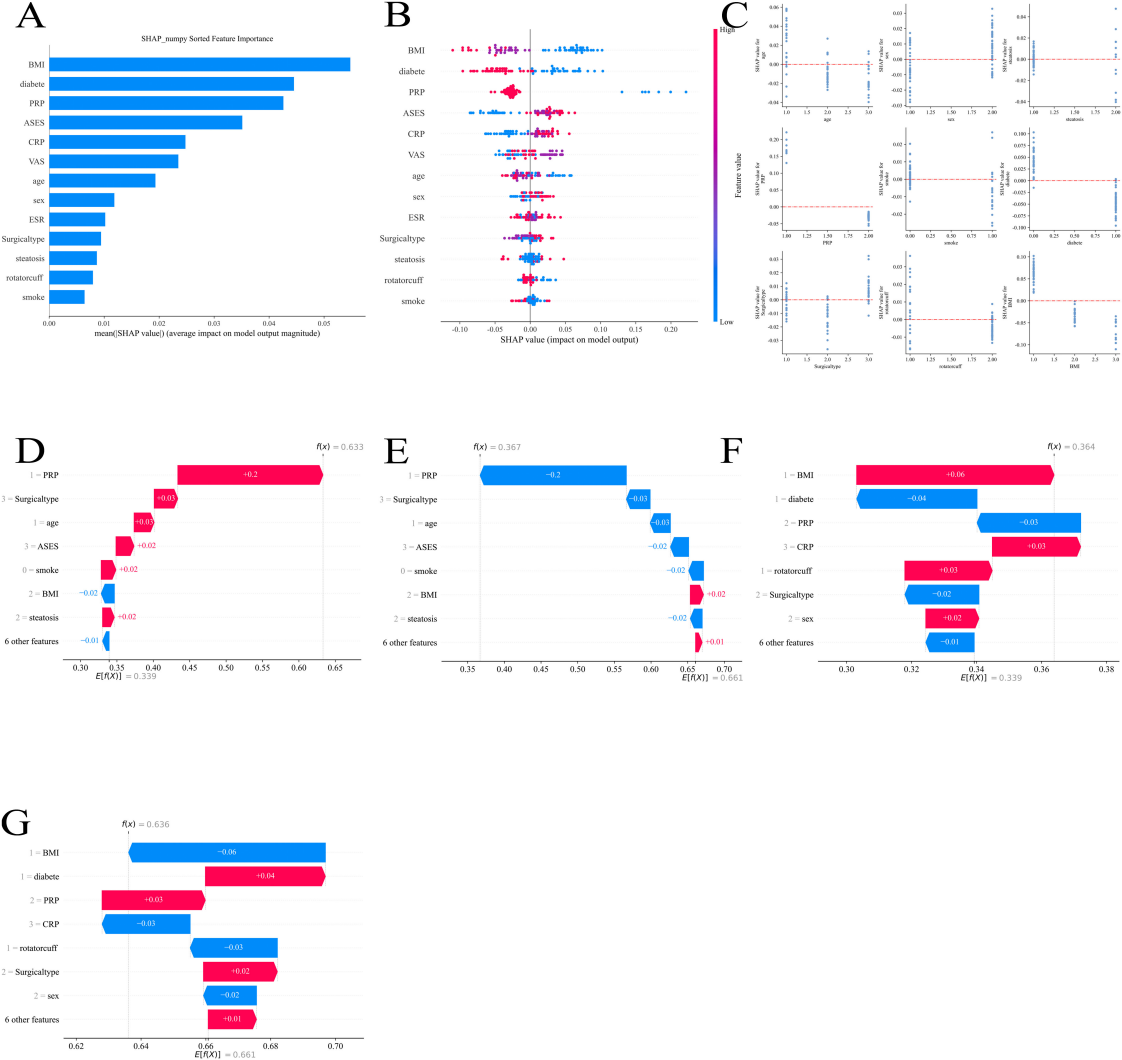
Shapley Additive Explanations (SHAP) analysis of feature importance and contribution to machine learning model predictions: **(A)** overall feature importance ranked by mean SHAP values; **(B)** SHAP summary plot showing feature-level contributions across patients; **(C)** SHAP dependence plots illustrating feature interactions and non-linear effects; **(D)** SHAP force plot example highlighting the contribution of, platelet-rich plasma (PRP) and other factors; **(E)** SHAP force plot example highlighting the contribution of surgical type and related features; **(F)** SHAP force plot example highlighting the contribution of body mass index (BMI), diabetes, and C-reactive protein (CRP); **(G)** SHAP force plot example showing combined feature effects including BMI, diabetes, PRP, CRP, and surgical type.

BMI (body mass index), diabetes, platelet-rich plasma (PRP), acromioclavicular function score (ASES), and C-reactive protein (CRP) had the biggest effects on the model’s predictive outcomes, according to the feature importance analysis in Figure 9A. This suggests that these factors are crucial for evaluating postoperative pain relief. In particular, PRP and ASES may be connected to the patient’s shoulder function and postoperative recovery, whereas BMI and diabetes have a greater place in the model and may be directly tied to the patient’s underlying health state.

Figure 9B demonstrates the distribution of SHAP values corresponding to each feature, further revealing the ways in which different features influence the predicted outcomes of the model. For example, BMI and diabetes had a large positive or negative effect on the predictive value of pain relief, and the different SHAP value intervals illustrate the different roles of these features in different patient groups.

By illustrating the relationships between characteristics, Figure 9C aids in our comprehension of how certain feature combinations affect prediction results. For instance, diabetes and BMI together may affect pain management differently, which gives us information on how to best tailor treatment plans.

The visual examination of feature contributions using various models is displayed in Figures 9D through 9G. These numbers demonstrate that PRP had a significant favorable impact in a number of models, suggesting that it is a significant predictor of postoperative pain alleviation. Furthermore, although their effects may be minimal, variables including age, ASES score, and kind of surgery also play a part in certain models.

## Discussion

The purpose of this study was to assess the impact of platelet-rich plasma (PRP) on postoperative pain relief after rotator cuff repair by combining nomogram modeling and machine learning. Additionally, we developed a personalized prediction model to help clinicians identify patients who would benefit most from PRP treatment. Optimizing pain management techniques and enhancing recovery results are the goals of this strategy.

We developed a prediction model that takes into consideration the intricate, non-linear connections between several variables affecting pain alleviation following surgery by fusing clinical data with cutting-edge machine learning techniques. Tree-based models like random forest (RF) and extreme gradient boosting (XGBoost) are excellent at capturing these complicated connections, but traditional statistical techniques like logistic regression frequently fail to adequately capture these complex relationships. In addition to increasing prediction accuracy, this method takes into account a variety of clinical and demographic characteristics that are frequently disregarded in conventional models, including age, BMI, smoking status, comorbidities, and baseline inflammatory markers.

Our findings indicate that PRP application is significantly associated with postoperative pain relief, consistent with existing clinical evidence suggesting that PRP promotes tendon healing and modulates inflammation (22–26). However, the effectiveness of PRP treatment was influenced by factors such as BMI, diabetes, and baseline inflammatory markers. These findings highlight the need for personalized treatment strategies. For example, patients with higher BMI tend to experience prolonged inflammation and altered pain sensitivity (27–29), potentially influencing both their baseline pain perception and their response to PRP treatment.

Using SHAP (Shapley Additive Explanations) analysis, we provided detailed insights into feature importance and their contributions to the model’s predictions. It was discovered that the combination of characteristics like diabetes and BMI significantly affected the results of pain alleviation, highlighting the necessity of tailored treatment strategies.

We created a nomogram that combines important indicators, such as preoperative pain levels, BMI, and PRP administration, giving physicians a simple, intuitive tool for forecasting pain alleviation results. The model’s robustness was further confirmed using calibration plots, which demonstrated a strong correlation between expected and actual results and proved the model’s dependability for clinical use.

Furthermore, we evaluated the performance of several machine learning models and discovered that, with higher AUC values, XGBoost and random forest performed well. This demonstrates how well these models work with complicated, high-dimensional data. However, models like the multilayer perceptron (MLP) and support vector machine (SVM) did not perform well, suggesting that not all machine learning models are appropriate for this particular prediction job.

The model’s capacity to distinguish between various patient groups was further validated by principal component analysis (PCA), underscoring the promise of machine learning in seeing patterns in clinical data that could be difficult to find with traditional techniques.

This study has some limitations, such as its retrospective methodology and possible biases in data collecting, despite its encouraging results. The model’s robustness will need to be confirmed by external validation in other cohorts, and more improvements are needed to enhance performance in more complicated situations or when several factors interact non-linearly. To improve the prediction effectiveness of the model, future studies should include more variables like genetic markers or more thorough imaging data.

To sum up, our work shows that postoperative pain alleviation after rotator cuff reconstruction may be predicted with machine learning algorithms and nomogram-based techniques, both of which are feasible and successful. We offer important insights into the function of biologic treatments in musculoskeletal surgery by including PRP administration as a significant predictor. This multidisciplinary approach represents a significant step toward precision medicine in orthopedic surgery, with the potential to enhance patient outcomes and optimize resource allocation, even though more validation and improvement are required.

The AdaBoost model showed a high training AUC (0.990) but a low test AUC (0.560), indicating potential overfitting. This is likely due to its sensitivity to noise and small sample sizes, suggesting that boosting may not be optimal for this clinical dataset.”

This study has limitations. Its retrospective single-center design may introduce selection bias and unmeasured confounding, despite strict eligibility criteria and multivariable adjustment. External validation on independent, multi-center datasets is still needed to confirm the robustness and generalizability of the model.

In summary, this study is the first to integrate biologically formulated PRP application with machine learning–based nomogram modeling to predict postoperative pain relief after rotator cuff repair. By combining clinical, demographic, and biological factors, our approach provides an innovative and interpretable tool for individualized pain management. This multidisciplinary integration of biomedicine and artificial intelligence not only enhances prediction accuracy but also offers practical guidance for clinical decision-making and future research.

## Conclusion

This study demonstrated that PRP administration significantly improves postoperative pain relief following rotator cuff repair. By integrating clinical variables and machine learning algorithms, a nomogram-based model was developed to assist personalized treatment planning. While the model showed promising performance, external validation and refinement are warranted to enhance generalizability.

## Data Availability

The raw data supporting the conclusions of this article will be made available by the authors, without undue reservation.
